# Distinguishing cultural experiences from psychotic symptoms in indigenous settings: Maori and North American perspectives

**DOI:** 10.1192/j.eurpsy.2021.846

**Published:** 2021-08-13

**Authors:** B. Mainguy, L. Mehl-Madrona, W. Nia Nia, A. Bush

**Affiliations:** 1 Education Division, Coyote Institute - Canada, Ottawa, Canada; 2 Medical Arts And Humanities Program, University of Maine, Orono, United States of America; 3 Cultural Component, Maori Mental Health Services, Wellington, New Zealand; 4 Psychiatric Services, Maori Mental Health Services, Wellington, New Zealand

**Keywords:** Indigenous culture, Maori, psychosis, Indigenous spirituality

## Abstract

**Introduction:**

Indigenous people think about mind and mental health differently from contemporary psychiatry, particularly in relation to the symptoms that comprise psychosis.

**Objectives:**

We aim to present the Maori (New Zealand) and the North American indigenous (primarily Lakota, Cherokee, and Wabanaki) views of extraordinary experience and to explore opportunities for dialogue and understanding among these perspectives, leading to genuine, respectful collaboration.

**Methods:**

Auto/ethnographic methodology was used to describe a process in which psychiatrists and traditional cultural healers came to understand each others’ perspectives, dialogued, and forged a collaboration. We describe how this process unfolded in New Zealand and in North America, discussing similarities and differences among these two regions and cultures. We present cases to illustrate the level of cultural collaboration.

**Results:**

The opportunity for cross-cultural dialogue arose when the psychiatrists observed that the traditional cultural healers were reaching and helping patients with whom they had been unsuccessful. This led to dialogue in the fashion of Two-Eyed Seeing, a North American indigenous concept of explanatory pluralism. We present the case of a young man whom the psychiatrist described as hallucinating and prescribed medication that did not help. The cultural healer assisted the young man to see how he had broken cultural taboos, helped him repair the damage, and the hallucinations disappeared. Other cases further illustrate the collaboration. Two-eyed seeing allows both perspectives to be correct and permits genuine dialogue.

**Conclusions:**

Through cultivation of genuine listening without interpretation or judgment (see Jacques Lacan), cultures can begin to understand and collaborate together for the benefit of patients.

